# The prevalence of falls and associated factors in older adults of the Torres Strait

**DOI:** 10.1111/ajag.13383

**Published:** 2024-10-27

**Authors:** Roselani Henry, Betty Sagigi, Gavin Miller, Sarah G. Russell, Fintan Thompson, Rachel Quigley, Edward Strivens

**Affiliations:** ^1^ Queensland Health Cairns and Hinterland Hospital and Health Service Cairns Queensland Australia; ^2^ Queensland Health Torres and Cape Hospital and Health Service Thursday Island Queensland Australia; ^3^ College of Medicine and Dentistry James Cook University Cairns Queensland Australia; ^4^ Australian Institute of Tropical Health and Medicine James Cook University Cairns Queensland Australia

**Keywords:** accidental falls, accidental injuries, Australian Aboriginal and Torres Strait Islander Peoples, healthy ageing

## Abstract

**Objective:**

To assess the prevalence of falls and examine associations between falls and potential risk factors in older adults of the Torres Strait Region of Australia.

**Methods:**

Two hundred and fifty people aged ≥45 years residing in the Torres Strait, who identified as Torres Strait Islander, Aboriginal or both, were asked whether they had sustained any falls in the past year. Associations between self‐reported falls and predictor variables were examined using logistic regression.

**Results:**

21% of participants reported at least one fall; 9% reported ≥2 falls. Participants who reported any falls in the past year were more than twice as likely to have urinary incontinence and poor mobility (*p* < .01) compared to participants who did not report any falls.

**Conclusions:**

Around one in five respondents reported one or more falls in the past year, demonstrating that falls are a significant issue for older adults of the Torres Strait. Fall prevention strategies that are effective in other populations are likely to be beneficial to the region but need to be informed by local consultation and implemented in partnership with the people of the Torres Strait.

**Practice Impact**

This study demonstrates that falls are a significant issue for older adults of the Torres Strait. The findings add to our understanding of falls in older First Nations Australians and provide a platform from which to advocate for the development of community‐driven fall prevention strategies.

## 1 INTRODUCTION

Falls are a significant cause of morbidity and mortality for older Australians and can lead to injury, functional decline and reduced participation in social activities.^1^, ^2^ Approximately one in three Australians aged 65 years and above report having had at least one fall in the past year.^3^, ^4^

Whilst recent studies have demonstrated that the prevalence of self‐reported falls in two Australian First Nations cohorts is comparable to this general rate, there is evidence that these falls are occurring at an earlier age, which significantly impacts quality of life in these younger cohorts.^5^, ^6^ Examining the reasons for falls in these younger groups is important to ensure appropriate programs are addressing specific risks within these communities.

A survey of 363 Aboriginal Australians aged 45 years and over living in the Kimberley region of Western Australia found that 31% of participants reported falling sometimes, and 12% reported having had an injurious fall.^5^ Factors associated with fall(s) included poor mobility, drinking alcohol, stroke, epilepsy, head injury and poor hearing.^5^ A follow‐up survey of 289 people from this cohort 5 years later found that 32% reported sustaining at least one fall in the past year.^7^

Another study, of 336 Aboriginal and/or Torres Strait Islander people aged 60 years and over living in urban and regional areas of New South Wales (NSW), found that 24% of participants reported at least one fall in the past year.^6^ Factors associated with fall(s) included being a woman, the use of three or more medications, having an arthritic condition, macular degeneration, depression, having a history of stroke, being unable to do one's own housework and being unable to do one's own shopping.^6^

The aim of this study was to examine falls among adults of the Torres Strait aged 45 years and over to inform relevant clinical responses. The Torres Strait Islands span the gap between the tip of Queensland's Cape York Peninsula and the southern coastline of Papua New Guinea. The region has been inhabited for thousands of years. Torres Strait Islanders have their own culture, languages and traditions.^8^ They are traditionally a seafaring, agriculturalist people with long‐term networks of connection and exchange with peoples of Papua New Guinea and mainland Australia.^8^, ^9^

In 2021, approximately 8390 people identifying as Torres Strait Islander, Aboriginal or both lived in the Torres Strait Islands and the region at the tip of Cape York, known as the Northern Peninsula Area. Approximately a quarter (23%) of this combined population were aged 45 years or older, an age at which problems associated with ageing have been shown to be present.^10^, ^11^

In an effort to inform culturally appropriate clinical geriatric and aged care services, the Queensland Health and James Cook University affiliated Healthy Ageing Research Team (HART) research team conducted an extensive dementia prevalence survey that included an assessment of rates and impact of age‐related conditions in people aged 45 years and over living in the Torres Strait.^11^ This paper reports rates of self‐reported falls in this group and evaluates associations between falls and several health and socio‐demographic factors.

## 2 METHODS

### 2.1 Ethics and governance

The study was co‐designed and conducted in partnership with the Post‐Acute, Rehabilitation and Aged Care Service on Thursday Island. Ethics approval was obtained from Queensland Health (HREC/13/QCH/129‐878) and James Cook University (H5495) Human Research Ethics Committees, which have specific processes for research conducted with Aboriginal and/or Torres Strait Island peoples.

This study came under the governance of an established Indigenous Reference Group (Knowledge Circle) who oversee all projects conducted by the team in the Torres Strait. The Knowledge Circle comprises First Nations academics, community members, aged care workers and health‐care staff who have expressed an interest in working with the research team on addressing health issues of adults in their communities. The Knowledge Circle provides expertise and guidance to ensure the research project methods and outcomes are culturally appropriate, take account of local issues and are undertaken in ways that promote capacity building. This project has been presented for discussion and approved by the Knowledge Circle prior to commencement.

### 2.2 The research team

The Healthy Ageing Research Team (HART) is a team of clinician researchers based in Far North Queensland who have been providing clinical aged‐care services to the Torres Strait for over two decades. The members of HART who conducted the research described here include a Torres Strait Islander health worker, an Aboriginal senior medical officer, and five non‐First Nations clinicians (a physiotherapist, two geriatricians and two neuropsychologists).

The Healthy Ageing Research Team (HART) is committed to community consultation, and we hope that our research will lead to tangible benefits to those involved. The data presented here were collected as part of a larger survey that was the product of several years of relationship‐building with communities of the Torres Strait. Members of HART built partnerships with the Aged Care Assessment Team and Post‐acute Rehabilitation and Aged Care service based on Thursday Island over years of clinical service. Both services collaborated on the design and implementation of this study, with one staff member formally joining the research team. Community members from several Island communities also contributed to the study design and data collection process. A reflection of HART experiences and lessons learned as a team of non‐First Nations and First Nations researchers in applying ethical approaches to research is described in Quigley et al.^12^

### 2.3 Study design and participants

This study has been guided by NHMRC guidelines including the 2018 *Ethical conduct in research with Aboriginal and Torres Strait Islander Peoples and communities: Guidelines for researchers and stakeholders* and *Keeping Research on Track II*.^13^, ^14^ Furthermore, the Aboriginal and Torres Strait Islander Quality Appraisal Tool (QAT) was used to assess the quality of the research from the perspective of First Nations Peoples (Table S1).^15^

The rolling prevalence study was undertaken from 2015 to 2018 in all populated islands and communities of the Torres Strait and Northern Peninsula Area of Cape York. Participants aged 45 years and over were recruited using lists from Primary Health Care Centres, and from community meetings and events attended by the research team. Additional recruitment arose from information distributed on community noticeboards and newsletters, local media and through word of mouth as described in Russell et al.^11^ The one nursing home in the Torres Strait was visited to ensure a heterogeneous sample of the community. There were no specific inclusion or exclusion criteria other than age. Participants were given written and verbal information about the study, and written consent was obtained.

Trained assessors collected data through structured face‐to‐face interviews with each participant, using the Kimberley Indigenous Cognitive Assessment (KICA) Tool. In some cases, interviews were also conducted with a participant's caregiver.

The KICA Tool was developed by researchers of the Western Australian Centre for Health and Ageing and provides a questionnaire for collecting social, functional, clinical and medical history. The KICA tool includes the Kimberley Indigenous Cognitive Assessment (KICA‐Cog),^16^ the KICA‐depression scale (i.e. a modified Patient Health Questionnaire [PHQ‐9])^17^ and the Elderly Falls Test (EFT).^18^ Minor modifications were made to the KICA tool. For example, in response to community advice, pictures used in the KICA‐Cog tool were changed from representations of cultural items and animals familiar to people of the Kimberley (e.g. boomerang, emu), to cultural items and animals familiar to people of the Torres Strait (e.g. headdress, cassowary). This modified KICA‐Cog has been validated in the Torres Strait.^19^

Participants also underwent a comprehensive assessment with a geriatrician, which included a person‐centred review of any chronic or emerging health concerns. A Torres Strait Islander member of the team provided cultural and language support for participants and advice for the research team throughout the survey. Assessments were completed at primary health centres, community halls or in participants' homes, depending on community and personal preferences.

Survey information was recorded on paper and later uploaded to a database. Audio recordings were not taken. De‐identified data were reviewed by a panel comprising geriatricians and an older person psychiatrist to obtain consensus diagnoses of cognitive status as described in Russell et al.^11^

### 2.4 Statistical analysis

Data were analysed using Stata 15 (StataCorp LLC, College Station, TX). The prevalence of past‐year fall was the per cent of participants who gave an answer to the first question in the older people fall screening test—‘How many times in the last year have you fallen down?’—that indicated one or more falls. Participants who reported at least one fall were then asked, ‘Did you hurt yourself?’. The prevalence rate of injurious falls was the per cent of people who answered ‘yes’ to this question.

For the purpose of analyses, participants were allocated to two groups: those who reported one or more past‐year falls, and those who reported no past‐year falls. Social, demographic and health characteristics were examined between the two groups using Pearson's *χ* ^2^ test for independence and Fisher's exact test to account for expected cell frequencies <5.^20^

Sixteen variables were selected for logistic regression analysis of association with past‐year fall. Variables were chosen using the published literature as a guide; they comprised some commonly cited risk factors and included factors that have been identified to have an association with falls in studies in other Australian First Nations communities.^5^, ^7^ The chosen variables are described in Appendix 1: Table A1.

To ensure that the regression analysis was appropriately powered, cross‐tabulations were performed between all possible combinations of discrete variables to assess for adequacy of expected frequencies.^17^ The variables ‘current smoking’, ‘stroke history’, ‘depression’ and ‘dependence of personal activities of daily living (pADLs)’ had cell sizes of less than 10 events when cross‐tabulated with falls in the past year. These variables were excluded from multivariate logistic regression analyses to maintain stability of the models.^21^

The variable ‘cognitive status’ was initially assessed as a categorical variable with three outcomes: normal, cognitive impairment no dementia (CIND) and dementia. However, when coded in this way, it did not meet adequacy of expected frequencies due to a relatively low number of people in the dementia category. For this reason, the variable was recoded to have a binary outcome, combining those people with CIND or dementia into the same category and comparing them to those with normal cognition.

Univariate logistic regression was performed separately for each of the remaining 12 independent variables and the dependent variable (past‐year fall). The assumption of linearity of the logit for the one continuous variable in the analysis (age) was confirmed via logistic regression of the interaction between age and its natural log with a Box–Tidwell regression model using the Stata command ‘boxtid’. The 12 independent variables were then entered into one multivariate logistic regression model, and variables with the least significance were removed over multiple rounds of backward removal using the Stata command ‘stepwise, pr(.1)’. In the final model, only variables that were significant at a threshold of *p* < .1 were retained. Due to missing data, the sample size for the multivariate analyses was reduced to 243 participants.

## 3 RESULTS

A total of 250 older adults who identified as Torres Strait Islander, Aboriginal or both, and who were living in the Torres Strait, were asked about falls sustained in the past year. The mean age of participants was 64 years (SD = 10.4, range 45–93).

Fifty‐four participants (22%) reported one or more falls in the year prior to the interview, with 22 (9%) reporting two or more falls. Thirty‐six participants reported having sustained an injury because of a fall, which was 14% of the total sample, or two‐thirds (67%) of participants who had sustained any falls.

Table 1 compares demographic, social and health characteristics between participants who reported at least one fall (*n* = 54) and those who reported no falls (*n* = 196). Participants who reported any falls in the past year were significantly more likely to report urinary incontinence (*p* = .004), poor mobility (*p* < .001), past stroke (*p* = .04) or dependence in instrumental activities of daily living (iADLs) (*p* = .03). All other study variables, including age and sex, were not associated with reports of falls in the past year.

**TABLE 1:** Demographic and clinical characteristics by status of falls in the past year, for 250 residents aged 45 years and older in the Torres Strait and Northern Peninsula Area of North Queensland, Australia (2015–2018).

Participant characteristics	Total (*n* = 250)		Past‐year fall(s) (*n* = 54)		No fall (*n* = 196)		*χ* ^2^ test	
	*n*	%	*n*	%	*n*	%	*χ* ^2^	*p*
Sex								
Women	163	65	35	65	128	65	.00	.95
Men	87	35	19	35	68	35		
Age (years)								
45–54	50	20	12	22	38	19	.51	.92
55–64	80	32	17	32	63	32		
65–74	71	28	16	30	55	28		
75–94	49	20	9	17	40	20		
Smoke cigarettes	39	16	8	15	31	16	.03	.86
Drinks alcohol (any amount)	67	27	12	22	55	28	.74	.39
Urinary incontinence	64	26	22	41	42	21	8.29	.004
Depression	17	7	6	12	11	6	1.99	.16
Poor mobility	76	30	27	50	49	25	12.51	.000
Poor vision	50	20	14	26	36	18	1.51	.22
Poor hearing	41	16	10	19	31	16	.23	.64
Pain	110	44	30	56	80	41	3.73	.05
Past stroke	13	5	6	11	7	4	4.88	.04
Diabetes	157	63	35	65	122	62	.12	.73
Cognitive status								
MCI (CIND)	52	21	18	33	34	17	7.64	.03
Dementia	28	11	3	6	25	13		
Dependence of pADLs	14	6	3	6	11	6	.00	.99
Dependence of iADLs	80	32	24	44	56	29	4.90	.03
Past head injury with LOC	44	18	11	21	33	17	.32	.57

Abbreviatoins: CIND, cognitive impairment not dementia; iADLs, independent activities of daily living; LOC, history of a hit to the head resulting in loss of consciousness; pADLs, personal activities of daily living.

Results of logistic regression analyses that examined the associations between 12 independent variables and the dependent variable of any falls in the past‐year are presented in Table 2. In univariate analyses, urinary incontinence and poor mobility were significantly associated with any falls in the past‐year and remained significant when all variables were entered into a single model. After backwards removal of variables that had least significance, participants who reported any falls in the past year were more than twice as likely to have urinary incontinence (OR = 2.49, 95%CI 1.28–4.85, *p* = .007) and poor mobility (OR = 2.86, 95%CI 1.50–5.45, *p* = .001) compared to participants who did not report any falls. Dementia or CIND was not associated with falls, and stroke was excluded from analyses due to small cell sizes.

**TABLE 2:** Associations between demographic and clinical characteristics with any falls in the past year, univariate and multivariate logistic regression with backward removal of variables, for 243 residents aged 45 years and older in the Torres Strait and Northern Peninsula Area of North Queensland, Australia (2015–2018).

Participant characteristics	Univariate regression			All variables regression			Backward removal^a^ (*p* < .100)		
	OR	95% CI	*p*	OR	95% CI	*p*	OR	95% CI	*p*
Sex (Reference: Women)									
Men	.98	.52, 1.84	.95	.94	.45, 1.98	.87			
Age (average one year change)	1.00	.97, 1.02	.77	.96	.93, 1.00	.05			
Drinks alcohol (any amount) (Reference: Never drinks alcohol)									
Yes	.73	.36, 1.49	.39	.70	.31, 1.58	.39			
Urinary incontinence (Reference: No or unsure)									
Yes	2.52	1.33, 4.79	.005	2.49	1.22, 5.10	.01	2.49	1.28, 4.85	.007
Poor mobility (Reference: No trouble walking)									
Yes	3.00	1.61, 5.60	.001	2.47	1.19, 5.13	.02	2.86	1.50, 5.45	.001
Poor vision (Reference: No poor vision or unsure)									
Yes	1.56	.77, 3.16	.22	1.81	.82, 4.01	.14			
Poor hearing (Reference: No poor hearing or unsure)									
Yes	1.21	.55, 2.66	.64	1.39	.55, 3.53	.49			
Pain (Reference: No or unsure)									
Yes	1.81	.99, 3.33	.06	1.66	.83, 3.31	.15			
Diabetes (Reference: No diabetes, pre‐diabetes or unsure)									
Yes	1.12	.60, 2.10	.73	.96	.47, 1.95	.90			
Cognitive status (Reference: No MCI (CIND) or dementia)									
MCI (CIND) or dementia	1.48	.79, 2.76	.22	1.65	.75, 3.62	.21			
Dependence of iADLs (Reference: Independent with iADLs)									
Yes	2.00	1.08, 3.72	.03	1.78	.83, 3.82	.14			
Past head injury with LOC (Reference: No or unsure)									
Yes	1.25	.58, 2.67	.57	.87	.37, 2.04	.75			

Abbreviations: CIND, cognitive impairment not dementia; iADLs, instrumental activities of daily living; LOC, history of a hit to the head resulting in loss of consciousness.

^a^ Backward removal of variables using a retainment threshold of *p* < .100.

Post hoc analyses showed urinary incontinence was more common among women (30%) compared to men (17%, *p* = .03). Consequently, the main analyses between falls and other study variables were repeated for men and women separately and reported in Table S2. Among men, any falls in the past year was significantly associated with increasing age (*p* = .01), dementia/CIND (*p* = .03) and dependence with iADLs (*p* = .04) after backwards removal regression, although some cells had less than 10 observations. The sub‐group analyses for women were comparable to the main analyses.

## 4 DISCUSSION

This study is the first analysis of falls and associated factors in older adults of the Torres Strait. Results show that falls are a significant issue, with one in five people reporting one or more falls in the past year. Two‐thirds of those who had fallen sustained an injury as a result of their fall, and people who reported falling were more likely to report concerns with urinary continence and mobility than people who had not fallen. The term ‘geriatric syndrome’ has evolved in the medical literature to describe those conditions that are common in older adults, do not fit into a discrete disease category and are multifactorial and leave a person vulnerable to stressors.^22^ Falling, urinary incontinence and poor mobility are geriatric syndromes. All are, in part, preventable and manageable. As such, there is potential for community‐driven healthy ageing strategies addressing these syndromes to bring meaningful quality‐of‐life improvements for older adults of the Torres Strait.

The prevalence of falls in this study (22%) is less than that reported in a similar study of First Nations Australians aged 45 years and older of the Kimberley region (32% of older adults in the Kimberley report falling in the past year^5^), but close to the rate of 24% reported by First Nations Australians aged 60 years and over in urban and regional NSW.^7^ Consistent with these studies, our study has also found that falls are occurring in younger age groups, highlighting the need for tailored fall prevention programs targeting this cohort.

In studies of general Australian populations using an age cut‐off of 65 years and older, roughly one in three people report a past‐year fall.^3^, ^4^ There are several possible reasons why the rate of falls in our study is lower than this. Risk of falling is influenced by health, environmental and social factors. The communities involved in this study included 18 island communities and five mainland communities in the Torres Strait and Northern Peninsula area, many of which are small in population number. It might be that there are protective social and environmental factors at play that benefit older adults in these communities. It may be that the population interviewed in this study is a less frail group of people than the general Australian over 65‐year‐old population. Additionally, there may be differences between groups in the likelihood of disclosing a fall that has been sustained. There are many reasons why an interviewee might choose not to report a fall. For example, they might be concerned of stigma, or of personal consequences that might arise from voicing vulnerability. Social factors are likely to play a part in how falls are perceived and reported, and these factors will vary between population groups.

Of the people participating in this study who reported a fall, two‐thirds reported having hurt themselves as a result. This is a reasonably high proportion when compared to other population studies in which—depending on the population and the definition of injury—it has been estimated that between 22% and 66% of older people who fall suffer an injury.^23^, ^24^ The high rate of fall‐related injury in this study is likely to include minor injuries such as bruises and abrasions, as well as more significant injuries.

In multiple regression analysis, participants who reported any falls in the past year were more than twice as likely to have urinary incontinence and poor mobility. These geriatric syndromes are recognised to have interrelated aetiology, with shared pathophysiological mechanisms and social and environmental influences. For example, an association between poor mobility—in our study defined as a self‐report of trouble walking—and falling has been demonstrated in other population groups.^24^ The two factors might even be seen as surrogate markers for the same entity: an instability or impairment of gait with reduced ability to navigate one's environment successfully. Urinary incontinence has also been established as an associated factor for falls in other studies, and shared predisposing factors for the two syndromes have been demonstrated.^25^, ^26^ By taking advantage of this interwoven causality, healthy ageing strategies could improve health outcomes across multiple syndromes.

Overall, our results suggest that the pathways that lead to falls in older adults are not vastly different in the Torres Strait than they are in other populations and that prevention strategies that are effective elsewhere, such as those described in the Australian Commission on Safety and Quality in Health Care's best practice guidelines for preventing falls,^27^ may be effective in the Torres Strait. However, it is vital that any programs intended to support First Nations people are community‐informed and culturally appropriate. Studies exploring the perspectives of older Aboriginal and Torres Strait Islander people of NSW on fall prevention and healthy ageing programs revealed a range of barriers to participation in mainstream programs, including lack of availability, transport constraints and a sense of disempowerment regarding healthy ageing.^28^, ^29^, ^30^ Participants felt that successful healthy ageing programs should address these barriers, allow for flexibility in attendance, foster social connections and be community‐specific/tailored and empower people with respect to ageing well.^28^, ^29^, ^30^ Elders viewed healthy ageing not as an absence of illness, but as a capacity to continue in roles of cultural leadership and as keepers of knowledge, spirituality and values.^30^ One example of a fall prevention program with strong community governance is the Ironbark Program, which has demonstrated feasibility and shown promising results for First Nations people of NSW.^31^ A similar strategy could be valuable in the Torres Strait, and it is recommended that any fall prevention programs for the Torres Strait are co‐designed with communities to ensure they reflect community wishes for how programs are to be developed and implemented.

### 4.1 Study limitations

The sample interviewed in this study represented approximately 17% of the First Nations population in the region aged 45 years and over.^11^ Whilst no participants declined to participate during recruitment, the sample may be biased towards those who were interested in attending for a health check and may not be representative of the wider community. Every effort was made to reach as many people as possible, and we hope to have achieved a reasonable cross‐section of the community. However, we cannot be certain that the study cohort is representative of the total population of older adults in the Torres Strait.

This study used self‐reported data, relying on recollection over the preceding year, with the consequent potential for recall bias. It has been demonstrated that 13% of older people with a fall in the past 12 months will not recall/report having had it when asked at the end of the time period.^32^ Furthermore, ‘fall’ was not strictly defined, so there may have been differing perceptions among respondents of what constituted a reportable incident. Survivor bias is also likely to influence the data; people who suffered death as a result of a fall, or who suffered fall‐related disability significant enough to necessitate a move to a larger centre in order to access care, would not have been included in the study. It is therefore likely that the true prevalence of falls in the Torres Strait is higher than estimated here.

Recall bias will have also affected many of the independent variables assessed for association with falls in the regression analyses, and several potentially significant falls risk factors could not be included. In some cases, this was because the necessary information was not collected in the survey. For example, information to calculate Body Mass Index was not collected in accordance with advice obtained through community consultation. The effect of polypharmacy on falls has been investigated in this sample and is reported elsewhere (under review). Finally, as discussed in the Methods section, the sample size of the study population limited the number of variables that could be included in the final analysis. Similarly, while our post hoc analyses suggested there were potential differences in the risks associated with falls between men and women, small cell sizes limited the interpretation of these findings.

## 5 CONCLUSIONS

Falls are a common problem for older adults living in the Torres Strait, with around one in five people reporting at least one fall in the past year. Geriatric syndromes associated with falling in this population include urinary incontinence and impaired mobility. Fall prevention strategies that are effective in other populations are likely to be beneficial for people of the Torres Strait and could form part of broader healthy ageing initiatives. To be successful, such strategies would need to be informed by local consultation, tailored to meet the needs of the community and implemented in partnership with the people of the Torres Strait.

## FUNDING INFORMATION

The project was funded by a three‐year NHMRC project grant (APP. 1106175).

## CONFLICT OF INTEREST STATEMENT

No conflicts of interest declared.

## Supporting information


Table S1.

Table S2.

## Data Availability

The data that support the findings of this study are available on request from the corresponding author. The data are not publicly available due to privacy or ethical restrictions.

## APPENDIX 1

**TABLE A1:** Definitions of demographic and clinical characteristics of residents aged 45 years and older in the Torres Strait and Northern Peninsula Area of North Queensland, Australia (2015–2018).

Study variable	Definition
Past year fall	Self‐report of one or more falls on the past year, compared to report of no past‐year falls
Age	Age in years
Sex	Man or woman
Alcohol Use	An answer of ‘yes’ to the question ‘Do you drink alcohol?’ compared to an answer of ‘no’
Cigarette smoking	An answer of ‘yes’ to the question ‘Do you smoke?’ compared to an answer of ‘no’
Urinary incontinence	An answer of ‘yes’ to the question ‘Do you ever leak urine?’ compared to an answer of ‘no’ or ‘don't know’
Depression	A score of ≥9 on the KICA‐depression (modified Patient Health Questionnaire) scale (i.e. a score indicating moderate to severe depression), compared to a score of <9
Poor mobility	An answer of ‘yes’ to the question ‘Do you have trouble walking?’ compared to an answer of ‘no’ or ‘don't know’
Poor vision	An answer of ‘no’ to the question ‘Are your eyes good? Can you see everything’ compared to an answer of ‘yes’ or ‘don't know’
Poor hearing	Hearing difficulties recorded from medical assessment and records
Pain	An answer of ‘yes’ to the question ‘De you ever have any pain?’ compared to an answer of ‘no’ or ‘don't know’
Stroke history	Cerebrovascular disease recorded from medical assessment and records
Diabetes	Type II diabetes recorded from medical assessment and records
Cognitive status	The presence of either MCI or dementia (as diagnosed by a Geriatrician, informed by CGA and cognitive assessments) compared to no MCI or dementia
Dependence of pADLs (personal activities of daily living)	Report of needing help with at least one of dressing and showering, compared to report of not needing help with either task (i.e. being partially or fully dependent on the task)
Dependence of iADLs (instrumental activities of daily living)	Report of needing help with at least one of cooking, cleaning, medication management and management of finances, compared to report of not needing help with any task (i.e. being partially or fully dependent on the task)
Past head injury with loss of consciousness	An answer of ‘yes’ to the question ‘have you ever been hit on the head and knocked out?’ compared to an answer of ‘no’ or ‘don't know’
