# Supplementary material for: The prevalence of falls and associated factors in older adults of the Torres Strait
Source: Australas J Ageing. 2024 Oct 27;44(1):e13383. doi: 10.1111/ajag.13383 (PMC11752826; doi:10.1111/ajag.13383)
Supplement: Supplementary file 1 — Table S1. [file AJAG-44-0-s001.docx]

# Supplementary Table 1

# ABORIGINAL AND TORRES STRAIT ISLANDER QUALITY APPRAISAL TOOL


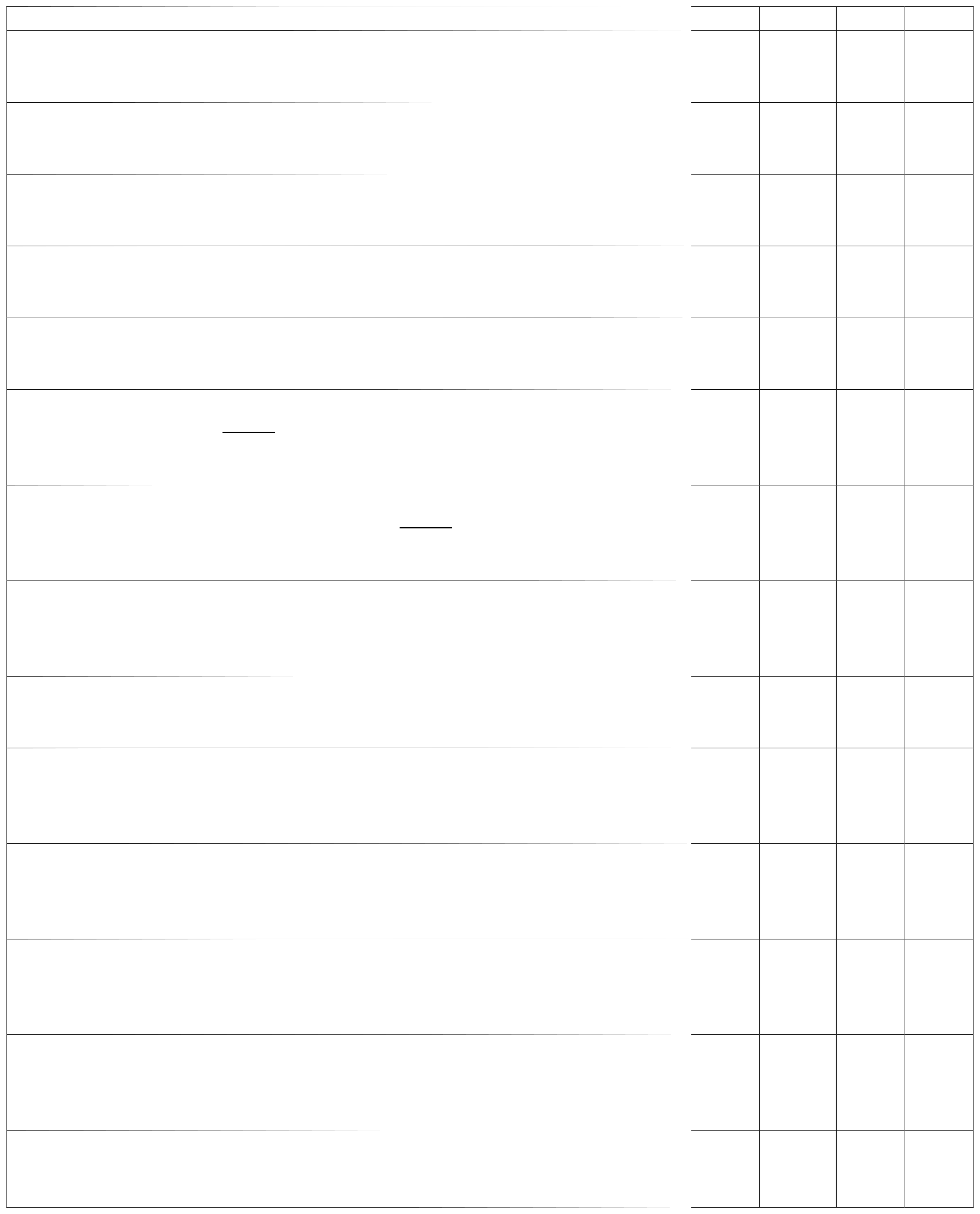

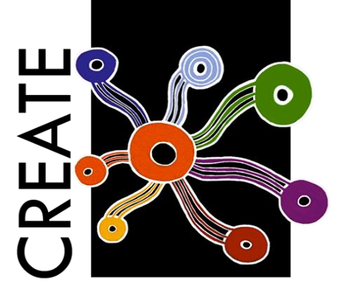

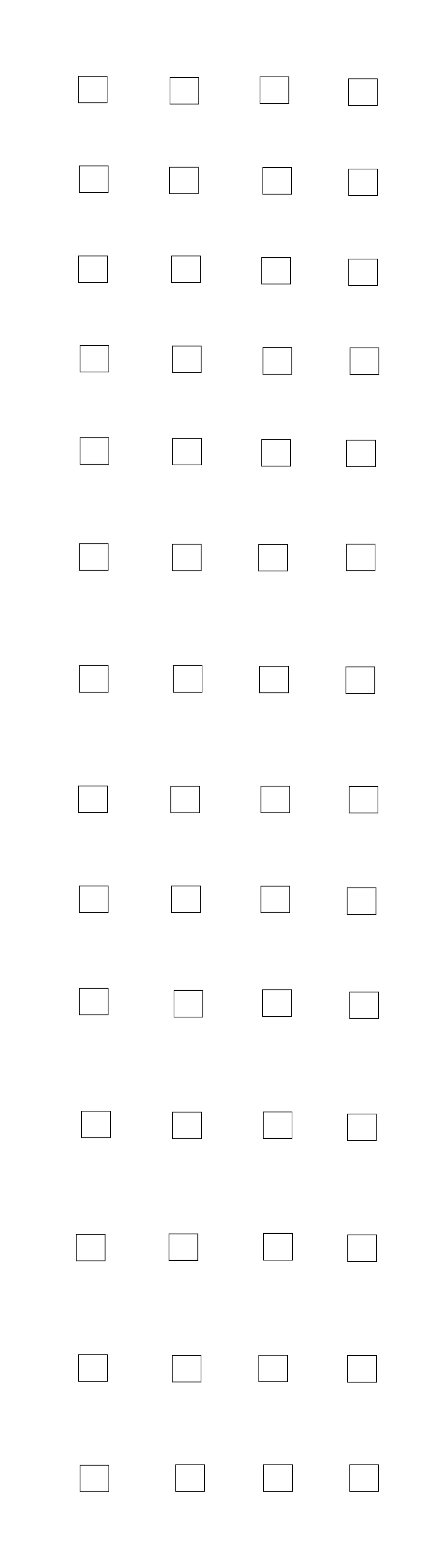


Answer either “Yes”, “Partially”, “No” or “Unclear” to each question

Article citation: The prevalence of falls and associated factors in older adults of the Torres Strait Reviewer’s name:___________________________

Question

1. Did the research respond to a need or priority determined by the community?

Yes

Partially X

No Unclear

1. Was community consultation and engagement appropriately inclusive?

# X

1. Did the research have Aboriginal and Torres Strait Islander research leadership?

# X

1. Did the research have Aboriginal and Torres Strait Islander governance?

# X

1. Were local community protocols respected and followed?

# X

1. Did the researchers negotiate agreements in regards to rights of access to Aboriginal and Torres Strait Islander peoples’ existing intellectual and cultural property?

# X

1. Did the researchers negotiate agreements to protect Aboriginal and Torres Strait Islander peoples’ ownership of intellectual and cultural property created through the research?

# X

1. Did Aboriginal and Torres Strait Islander peoples and communities have control over the collection and management of research materials?

# X

1. Was the research guided by an Indigenous research paradigm?

# X

1. Does the research take a strengths-based approach, acknowledging and moving beyond practices that have harmed Aboriginal and Torres Strait peoples in the past?

# X

1. Did the researchers plan and translate the findings into sustainable changes in policy and/or practice?

# X

1. Did the research benefit the participants and Aboriginal and Torres Strait Islander communities?

# X

1. Did the research demonstrate capacity strengthening for Aboriginal and Torres Strait Islander individuals?

# X

1. Did everyone involved in the research have opportunities to learn from each other?

X
