# Supplementary material for: The prevalence of falls and associated factors in older adults of the Torres Strait
Source: Australas J Ageing. 2024 Oct 27;44(1):e13383. doi: 10.1111/ajag.13383 (PMC11752826; doi:10.1111/ajag.13383)
Supplement: Supplementary file 2 — Table S2. [file AJAG-44-0-s002.docx]

**Supplementary Table 2:** Associations between demographic and clinical characteristics with any falls in the past year, for men (n=87) and women (n=163) separately, with backward removal of variables, residents aged 45 years and older in the Torres Strait and Northern Peninsula Area of North Queensland, Australia (2015-2018)

| **Participant Characteristics** | **Men (n=87)** | | | | | | | |  | **Women (n=163)** | | | | | | | |
| --- | --- | --- | --- | --- | --- | --- | --- | --- | --- | --- | --- | --- | --- | --- | --- | --- | --- |
|  | **Past-year fall(s)  (n=19)** | | **No fall  (n=68)** | | **χ^2^ test** | **Backward removal (*p*<0.100)** | | |  | **Past-year fall(s)  (n=35)** | | **No fall  (n=128)** | | **χ^2^ test** | **Backward removal (*p*<0.100)** | | |
|  | **n** | **(%)** | **n** | **(%)** | ***p*** | **OR** | **(95% CI)** | ***P*** |  | **n** | **(%)** | **n** | **(%)** | ***p*** | **OR** | **(95% CI)** | ***P*** |
| Age (years) |  |  |  |  |  |  |  |  |  |  |  |  |  |  |  |  |  |
| 45-54 | 5 | (26) | 15 | (22) | 0.32 | 0.90 | 0.83, 0.97 | 0.01 |  | 7 | (20) | 23 | (18) | 0.85 |  |  |  |
| 55-64 | 4 | (21) | 21 | (31) |  |  |  |  |  | 13 | (37) | 42 | (33) |  |  |  |  |
| 65-74 | 8 | (42) | 16 | (24) |  |  |  |  |  | 8 | (23) | 39 | (31) |  |  |  |  |
| 75-94 | 2 | (11) | 16 | (24) |  |  |  |  |  | 7 | (20) | 24 | (19) |  |  |  |  |
| Drinks alcohol | 7 | (37) | 25 | (37) | 0.995 |  |  |  |  | 5 | (14) | 30 | (23) | 0.24 |  |  |  |
| Urinary Incont. | 6 | (32) | 9 | (13) | 0.09 | 3.50 | 0.84, 14.59 | 0.09 |  | 16 | (46) | 33 | (26) | 0.02 | 2.47 | 1.10, 5.54 | 0.03 |
| Poor mobility | 9 | (47) | 17 | (25) | 0.09 |  |  |  |  | 18 | (51) | 32 | (25) | 0.003 | 3.41 | 1.54, 7.57 | 0.003 |
| Poor vision | 6 | (32) | 11 | (16) | 0.19 |  |  |  |  | 8 | (23) | 25 | (20) | 0.66 |  |  |  |
| Poor hearing | 8 | (42) | 18 | (27) | 0.26 | 3.68 | 0.92, 14.70 | 0.07 |  | 2 | (6) | 13 | (10) | 0.53 |  |  |  |
| Pain | 10 | (53) | 29 | (43) | 0.44 |  |  |  |  | 20 | (57) | 51 | (40) | 0.07 |  |  |  |
| Diabetes | 11 | (58) | 38 | (56) | 0.88 |  |  |  |  | 24 | (69) | 84 | (66) | 0.74 |  |  |  |
| Cognitive status |  |  |  |  |  |  |  |  |  |  |  |  |  |  |  |  |  |
| Normal | 9 | (47) | 49 | (72) | 0.06 | 6.72 | 1.19, 37.76 | 0.03 |  | 24 | (69) | 88 | (69) | 0.98 |  |  |  |
| Dementia/MCI | 10 | (53) | 19 | (28) |  |  |  |  |  | 11 | (31) | 40 | (31) |  |  |  |  |
| Depend. iADLs | 10 | (53) | 17 | (25) | 0.03 | 4.83 | 1.08, 21.59 | 0.04 |  | 14 | (40) | 39 | (31) | 0.29 |  |  |  |
| LOC | 4 | (22) | 13 | (20) | 0.75 |  |  |  |  | 7 | (20) | 20 | (16) | 0.61 |  |  |  |

Notes: CIND = Cognitive Impairment Not Dementia, Depend iADLs = Partial or full dependence with Instrumental Activities of Daily Living, LOC = History of a hit to the head resulting in loss of consciousness, Urinary Incont.= Urinary incontinence. Backward removal of variables using a retainment threshold of *p*<0.100, with Age treated as a continuous variable in regression models.
